# My stomach is full

**DOI:** 10.1192/j.eurpsy.2022.1494

**Published:** 2022-09-01

**Authors:** M. Queipo De Llano De La Viuda, A. Gonzaga Ramírez, N. De Uribe Viloria, G. Guerra Valera, T. Jiménez Aparicio, C. Vallecillo Adame, C. De Andrés Lobo, I. Santos Carrasco, J. Gonçalves Cerejeira, N. Navarro Barriga, M.J. Mateos Sexmero, B. Rodríguez Rodríguez, M. Fernández Lozano

**Affiliations:** 1 Hospital Clínico Universitario de Valladolid, Psychiatry, Valladolid, Spain; 2 Hospital Universitario Fundación de Alcorcón, Psychiatry, Madrid, Spain

**Keywords:** bezoar, anorexia, Trichotillomania, trichophagia

## Abstract

**Introduction:**

Anorexia nervosa is an eating behavior disorder that is often related to various personality factors. The relationship between obsessive compulsive disorder and eating Disorders has been highlighted.

**Objectives:**

To present a clinical case of a patient with eating disorder and gastric bezoar, secondary to compulsive hair ingestion.

**Methods:**

Bibliographic review of articles published in relation to the comorbidity of these disorders, based on articles published in the last 5 years in Pubmed.

**Results:**

26-year-old female. Diagnosis of restrictive anorexia nervosa. She was admitted to the hospital on two occasions for nutritional disorders. In the last admission, she reported greater anxiety and significant weight loss. She reports that she has limited her food intake, but she does feel thin and is unable to eat for fear of gaining weight. Ruminative thoughts about her body image. During admission, the patient expressed a sensation of fullness, nausea and vomiting, later observing in abdominal X-ray and gastroscopy, the presence of a gastric trichobezoar, which was finally resolved conservatively.

**Conclusions:**

Trichotillomania is observerd in 1 in 2000 people, trichophagia is even less frequent. According to DSM- V, these disorders are grouped within obsessive-compulsive spectrum disorders. A Trichobezoar is a conglomerate that can be found in the stomach or intestine, composed mainly of hair, previously ingested. Trichotillomania can be associated with anorexia nervosa, especially in patients with obsessive personality traits, which occurs frequently. The gastric slowing that patients with anorexia often present is a factor that favors the formation of the bezoar

**Disclosure:**

No significant relationships.

